# Three dimensional evaluation of cerebrovascular density and branching in chronic traumatic encephalopathy

**DOI:** 10.1186/s40478-023-01612-y

**Published:** 2023-07-25

**Authors:** Grace Rosen, Daniel Kirsch, Sarah Horowitz, Jonathan D. Cherry, Raymond Nicks, Hunter Kelley, Madeline Uretsky, Kevin Dell’Aquila, Rebecca Mathias, Kerry A. Cormier, Caroline A. Kubilus, Jesse Mez, Yorghos Tripodis, Thor D. Stein, Victor E. Alvarez, Michael L. Alosco, Ann C. McKee, Bertrand R. Huber

**Affiliations:** 1 VA Boston Healthcare System, US Department of Veterans Affairs, 150 S Huntington Avenue, Boston, MA 02130 USA; 2 Department of Pathology and Laboratory Medicine, Boston University Chobanian & Avedisian School of Medicine, Boston, USA; 3 Department of Neurology, Boston University Chobanian & Avedisian School of Medicine, Boston, USA; 4 Boston University Alzheimer’s Disease Research Center and Boston University CTE Center, Boston, USA; 5 VA Bedford Healthcare System, US Department of Veterans Affairs, Bedford, MA USA; 6 National Center for PTSD, US Department of Veterans Affairs, Boston, MA USA; 7 Department of Biostatistics, Boston University School of Public Health, Boston, USA

**Keywords:** Traumatic brain injury, Chronic traumatic encephalopathy, Neurodegeneration, Cerebrovascular, Tissue clearing, Clarity, Fluorescent microscopy

## Abstract

Chronic traumatic encephalopathy (CTE) is a neurodegenerative disease associated with exposure to repetitive head impacts (RHI) and characterized by perivascular accumulations of hyperphosphorylated tau protein (p-tau) at the depths of the cortical sulci. Studies of living athletes exposed to RHI, including concussive and nonconcussive impacts, have shown increased blood–brain barrier permeability, reduced cerebral blood flow, and alterations in vasoreactivity. Blood–brain barrier abnormalities have also been reported in individuals neuropathologically diagnosed with CTE. To further investigate the three-dimensional microvascular changes in individuals diagnosed with CTE and controls, we used SHIELD tissue processing and passive delipidation to optically clear and label blocks of postmortem human dorsolateral frontal cortex. We used fluorescent confocal microscopy to quantitate vascular branch density and fraction volume. We compared the findings in 41 male brain donors, age at death 31–89 years, mean age 64 years, including 12 donors with low CTE (McKee stage I–II), 13 with high CTE (McKee stage III–IV) to 16 age- and sex-matched non-CTE controls (7 with RHI exposure and 9 with no RHI exposure). The density of vessel branches in the gray matter sulcus was significantly greater in CTE cases than in controls. The ratios of sulcus versus gyrus vessel branch density and fraction volume were also greater in CTE than in controls and significantly above one for the CTE group. Hyperphosphorylated tau pathology density correlated with gray matter sulcus fraction volume. These findings point towards increased vascular coverage and branching in the dorsolateral frontal cortex (DLF) sulci in CTE, that correlates with p-tau pathology.

**Supplementary Information:**

The online version contains supplementary material available at 10.1186/s40478-023-01612-y.

## Introduction

Chronic traumatic encephalopathy (CTE) is a progressive neurodegenerative tauopathy caused in part by exposure to repetitive head impacts (RHI), such as those experienced through contact sport participation, military combat, and physical violence [49, 51, 54, 67]. RHI are a form of multiple brain injuries that include symptomatic concussion, characterized by symptoms such as headache, anxiety, cognitive impairment, and sleep disturbances, and non-concussive asymptomatic head impacts. A definitive diagnosis of CTE requires post-mortem neuropathological exam and the presence of the pathognomonic lesion, a perivascular accumulation of hyperphosphorylated tau (p-tau) aggregates in neurons, with or without astrocytic p-tau inclusions, at the depths of the cortical sulci [11, 50]. In 2013, McKee and colleagues proposed a staging scheme to characterize the severity of the p-tau pathology in CTE, stages I–IV, based on the density and regional deposition of p-tau pathology [4, 8].

Several studies have demonstrated a dose–response relationship between the duration of exposure to RHI and risk for CTE and CTE severity [4, 51, 53, 54, 56, 57]. In addition, the location of the diagnostic perivascular lesions of CTE at the sulcal depths of the frontal cortex parallels the areas of greatest tissue deformation and strain in computational and finite element models of helmeted head impact injury [14, 21, 83]. Exposure to RHI and CTE are also associated with increases in neuroinflammation that parallels the severity of p-tau pathology and correlates with years of RHI exposure [18].

Vascular dysfunction and injury have been observed after mild traumatic brain injuries (mTBI), RHI, and in CTE. Breakdown of the blood–brain barrier has been shown in the acute period in TBI-exposed mice [10], after mTBI in rodent models [28, 36, 71, 80, 81], and in the context of RHI in football players [75]. Reduced blood–brain barrier permeability regulator levels have been found in regions of high p-tau pathology in an individual with pathologically verified CTE [24] and in the serum of military personnel after acute repetitive blast exposure [2]. Markers of vascular injury were increased in the dorsolateral frontal cortex (DLF) of individuals with CTE [38]. Vascular dysfunction in rodent models includes reduced meningeal lymphatic drainage up to at least one month-post TBI [13] and reduced vasoreactivity after mTBI and RHI [31, 47]. In addition, decreased cerebral blood flow has been observed after single mTBI exposure in mice [32, 33, 79], RHI exposure in mice [1, 46], mTBI patients [42], and in elite rugby players [84].

Cerebrovascular structural changes have also been observed in mTBI, RHI, and CTE. Twenty-four hours after mTBI in rodents, microscopic changes can be seen, including microvascular degeneration, vascular cell apoptosis [37], and decreased vessel density compared to controls [45]. In the post-acute period in mouse models, cerebrovascular volume is increased after exposure to RHI [1] and mTBI [34, 80]. In humans, traumatic microbleeds can be found after acute TBI [30], and individuals with CTE show a propensity toward comorbid arteriolosclerosis [7]. RHI exposure has been associated with increased white matter hyperintensities on FLAIR magnetic resonance imaging (MRI), possibly due to microvascular injury [5, 74]. Microvascular abnormalities have also been observed in postmortem brain of individuals with CTE [7, 52, 53, 69]. Studies of the chronic effects of repetitive mTBI on vascular morphology in human subjects have demonstrated an expanded perivascular space around large caliber vessels on MRI [64]. Given the evidence for vascular structural changes, functional alterations and cognitive impairment in rodent models [42, 46, 78] and vascular comorbidities in human subjects with CTE, we sought to investigate the vascular changes in CTE DLF cortex using fluorescent microscopy and three-dimensional (3-D) imaging of blood vessel morphology within optically cleared tissue blocks.

Light microscopic studies are limited in detecting and quantifying morphologic changes in brain capillaries due to the sparse sampling of vascular cross-sections in two dimensions. In contrast, tissue clearing methods are well suited for characterizing small 3-D structures in the brain, as light scattering is minimized and vascular branching can be assessed using 200 µm tissue sections. Electrophoresis-driven clearing and fluorescent labeling have been successfully employed to image whole mouse brains [19, 37, 82] and many human organs, including the kidney, pancreas, heart, lung, spleen, and brain [48, 68]. Previous studies clearing postmortem human brain sections have either required minimal fixation, several months of clearing [20, 41, 43, 44, 60, 63], or the use of 100 µm sections cleared over a few weeks that warped the tissue surface [61]. The utility of tissue clearing has been demonstrated through the qualitative visualization of Alzheimer's Disease (AD) plaques in the human brain [8]. Recently, SHIELD postfixation has been developed for single cell three-dimensional microscopy with various methods of tissue clearing [62].

Here, we applied SHIELD, tissue clearing and staining protocols over 2 weeks using 200 µm thick slices of postmortem human brain to visualize DLF capillary morphology in brain donors with and without CTE.

## Materials and methods

### Study design and brain donors

Autopsy participants included 41 male brain donors from three brain banks housed at VA Boston Healthcare System with harmonized neuropathological processing protocols and diagnostic procedures: Understanding Neurological Injury and Traumatic Encephalopathy (UNITE, n = 31), Boston University Alzheimer's Disease Research Center (ADRC, n = 1) and the National Posttraumatic Stress Disorder Brain Bank (PTSD, n = 9). For this study, participants were categorized based on their CTE diagnosis into two groups: "non-CTE" (*n* = 16) and *"*CTE" (high and low severity), *n* = 25. Participants were excluded if there was neuropathological evidence of co-morbid neurodegenerative disease [i.e. Alzheimer's disease, Lewy body disease, amyotrophic lateral sclerosis, significant vascular disease (including infarcts, microinfarcts, and lacunes in the DLF) or causes of death that might affect tissue integrity (e.g., gunshot wounds to the head, drowning) as well as for missing primary study variables (CTE severity, CAA status)]. Participants with arteriolosclerosis and/or mild to moderate atherosclerosis were included and adjusted for statistically. Participants' causes of death and comorbidities are listed in Additional file 1: Table S1. The donors' next of kin provided consent for brain donation and research participation. Institutional review boards from the Boston University Medical Center approved brain donation, postmortem clinical record review, neuropathological evaluation, and clinical interviews with donor family members.

### Clinical assessment and diagnosis

Demographic information, medical history, and other antemortem clinical variables were obtained during retrospective clinical evaluation with informants for all brain donors and included a detailed assessment of RHI exposure, including sports played, primary sport, position, age at first exposure, years played, concussion history, and all other TBI history [58].

### Tissue processing and pathological assessment

Postmortem brain tissue was fixed in periodate-lysine-paraformaldehyde (PLP) for at least 3 months at 4 °C. The neuropathological assessment was performed using procedures previously established [58, 76]. Neuropathological evaluations were made by board-certified neuropathologists (ACM, TDS, BRH) according to published diagnostic criteria and were kept blinded to antemortem clinical information [58]. Cerebral arteriolosclerosis, atherosclerosis, and CAA were evaluated on a semiquantitative scale [12, 73].

For immunohistochemical assessment, 20 µm slides from paraffin-embedded tissue blocks from the DLF were prepared and stained for AT8 as previously described [16]. Slides were scanned, digitized at 20× magnification, and analyzed for AT8 density (total area in the sulcus positive for AT8 staining divided by the total area of tissue analyzed) using Aperio Scanscope (Leica) as previously described [17]. The depth of the cortical sulcus was defined as the bottom third of two connecting gyri. Ki67 staining was completed using Leica’s Ready-to-Use Ki67 antibody reagent on the BOND staining system (Leica, Deer Park, IL) and imaged on the Vectra Polaris slide scanner (Akoya, Marlborough, MA).

### Passive tissue clearing and labeling

**Fig. 1:**
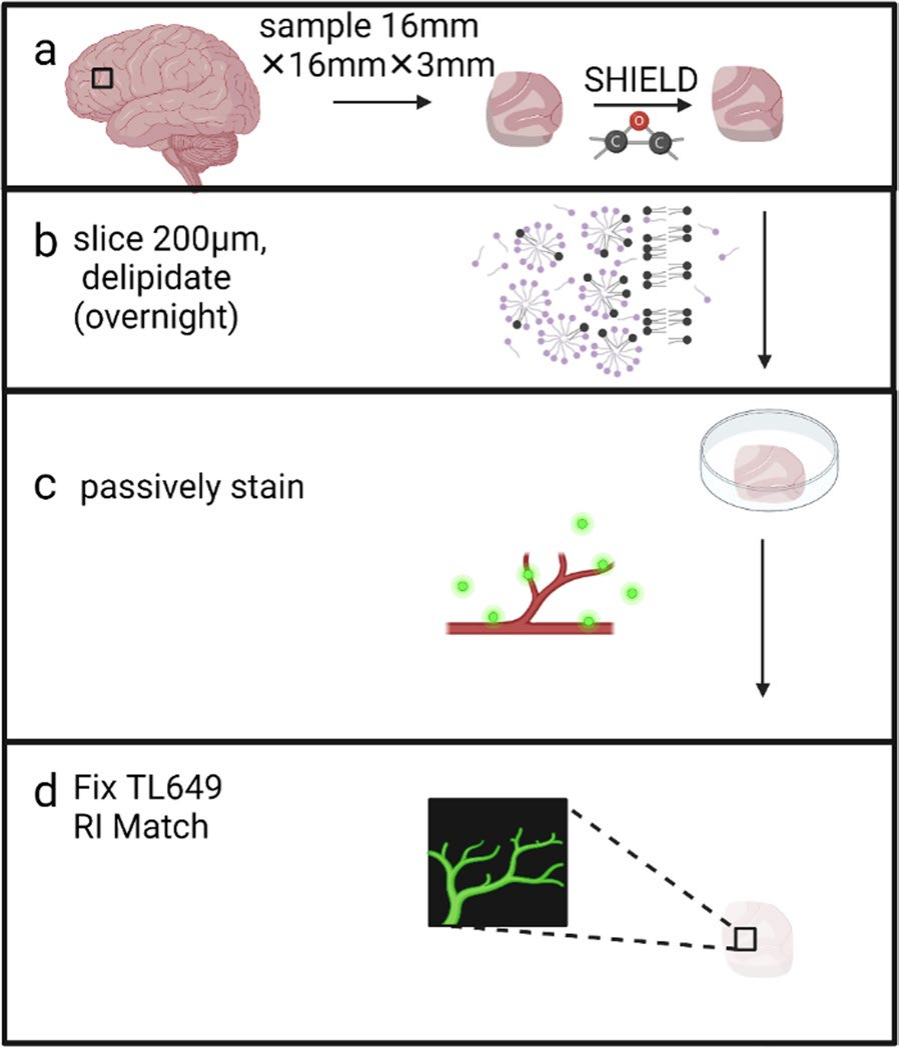
Workflow for tissue clearing and staining. **a** Before the clearing process, tissue is prepared for clearing by cross-linking proteins and nucleic acids using epoxides. This cross-linking provides increased structural stability in preparation of lipid removal from the tissue [62]. Lipids are washed from the tissue using sodium dodecyl sulphate (SDS). The fully cleared tissue can be fluorescently labeled and incubated in an index of refraction matching solution so that its refractive index matches that of a glass cover-slip. **b** Passive tissue clearing is diffusion based using a floating sections protocol. **c** Fluorescent molecules diffuse to their target sites in the floating section. **d** After the fluorescent dyes have been fixed in place and the sample has been refractive-index (RI) matched to the glass coverslip, the tissue becomes translucent. Fluorophores can be visualized at any point in the Z-dimension of the respective samples. Created with www.BioRender.com

PLP-fixed DLF (Brodmann area 46) tissue blocks were harvested with a 16mm^2^ leather punch with a thickness of 3 mm. Tissue harvesting was performed blinded to CTE status based on a standardized blocking scheme that is used in al cases. The 16 × 16 × 3 mm tissue sections were incubated in SHIELD OFF solution (LifeCanvas SHIELD-buffer solution, LifeCanvas SHIELD-Epoxy solution, and deionized water) for three days with shaking at 4 °C, transferred to SHIELD ON buffer and incubated for 24 h with shaking at 37 °C, and stored in phosphate-buffered saline (PBS) with 0.02% sodium azide for up to several weeks. SHIELD-preserved sections were embedded in 2% agarose gel and cut into 200 µm slices using a vibratome (VF-700-0Z Microtome, Precisionary Instruments, Inc, Natick, MA). Slices were stored in PBS with 0.02% sodium azide. To delipidate, slices were incubated overnight at 37 °C with light shaking in passive clearing buffer [300 mM sodium dodecyl sulfate (SDS), 10 mM boric acid, 100 mM sodium sulfite, pH 9]. Slices were subsequently washed twice with PBST (PBS + 0.4% Triton-X) at room temperature with light shaking to wash out the SDS then photobleached for five days in PBST as previously described [40]. Slices were incubated with 40 µg tomato lectin^649^ in 1 mL of PBST for 24 h with light shaking. Samples were washed three times with PBST for 30 min and incubated overnight in 4% PFA (Affymetrix) to post-fix the dye to the tissue. The next day the samples were washed three times, two hours per wash, in PBS. Samples were incubated overnight in EasyIndex (LifeCanvas) before mounting onto a 25 × 75 × 1.0 mm glass slide (Fisher, Pittsburgh, PA) and covered with a No. 1 22 × 30 mm glass coverslip (Corning GlassWorks, Corning, NY). Imaging was performed with a Nikon Eclipse Ti2-E spinning disk confocal microscope and Nikon Elements software (Nikon, Melville, NY) (Fig. 1).

### Imaging of cleared tissue

Tomato lectin^649^ was excited using the 633 nm laser line of a Celesta light source, at 72.8% power with an exposure time of 400 ms. A Plan Apo λ 20 × air objective (Nikon, Melville, NY) was used to acquire up to four regions each in the gray matter crest, gray matter sulcus, white matter crest, and white matter sulcus. Sulcus imaging regions were randomly selected in the bottom third of two connecting gyri, and crest imaging regions were randomly selected in the top third of the gyrus. For each image stack, image slices were taken 0.9 µm apart. To capture the highest resolution images, we used a Plan Apo λ 60 × oil-immersion objective (Nikon, Melville, NY). Tissue imaging and image analysis were performed blinded to CTE status.

### Quantification of blood vessels and variables measured using fluorescence microscopy

Passively cleared DLF samples were quantified using Imaris 9.6 and 9.7 (Oxford Instruments, Abington, Oxfordshire UK), (Table 1). For each image, a surface was generated in Imaris using the Surface Tool to represent blood vessels based on fluorescent intensity and volume. Pixels outside of the generated surface were set to zero to eliminate non-specific background fluorescence. The volume of that surface divided by the total volume of the image stack represented the unitless fraction volume calculation. Blood vessel branches were drawn manually using the Imaris Filament Tool. The total number of drawn filament segments was divided by the total volume of the image stack to calculate branch density in units of branches/µm^3^. For each case, the fraction volume and branch density measurements were an average of the measurements made from the replicate image stacks per region.

**Table 1:** Summary of variables used to quantify vascular density and branching

Measured variable	Calculation
Branch Density (branches/µm^3^)	Number of branches/volume of image stack
Fraction Volume (unitless)	Volume of blood vessels/volume of image stack
Gray Matter Branch Density Ratio (unitless)	Gray matter sulcus branch density/gray matter crest branch density
Gray Matter Fraction Volume Ratio (unitless)	Gray matter sulcus fraction volume/gray matter crest fraction volume
White Matter Branch Density Ratio (unitless)	White matter sulcus branch density/white matter crest branch density
White Matter Fraction Volume Ratio (unitless)	White matter sulcus fraction volume/white matter crest fraction volume

In order to quantify differences in vascular morphology, we quantified blood vessel branch density via the number of blood vessel branches per unit volume. We used blood vessel fraction volume, the fraction of the image volume composed of blood vessels, to quantify vascular coverage. Because the pathognomonic CTE lesion is in sulcal depths, we compared measurements in the sulcus vs the gyrus. Similarly we correlated the vascular fraction volume and branch density with p-tau pathology to determine if p-tau accumulation colocalized with altered vascular morphology.

### Statistical analyses

The fluorescence microscopy data were analyzed using SPSS (v.27, IBM, Inc, Armonk, NY) and GraphPad Prism (v.9.0.0, GraphPad Software, La Jolla, CA). An analysis of covariance (ANCOVA) was used to compare fraction volume or branch density changes among control and CTE groups. Age at death was included in all analyses as a covariate to control for age-associated differences. Arteriolosclerosis and atherosclerosis were not included as covariates, as the F-statistic was not significant for any measured variables. Cerebral amyloid angiopathy (CAA) was also included as a covariate. Associations among p-tau burden and vascular variables were measured with multiple linear regression models with p-tau staining density, age of death, and CTE status as the independent variables and gray matter vessel fraction volume or branch density as the dependent variable. Cases with p-tau staining data were those with CTE (*n* = 22) and a subset of non-CTE controls (*n* = 8). Descriptive statistics were generated with SPSS. The significance level was set a priori to 5%. Statistical power was calculated with MATLAB 2020a’s samplesizepwr function. The full dataset with clinical, neuropathological, and measured vascular variables can be found in Additional file 2.

## Results

### Demographic statistics of experimental groups

Experimental groups were all male did not differ in mean age at death (Table 2). Individual participants may have more than one RHI exposure source.

**Table 2:** Donor demographic statistics

	Controls	CTE	*p*-Value
Sample size (n)	16	25	
Age at death (S.E.M), range, years	65 (3), 46–86	62 (4), 31–89	*P* = 0.694
Race or ethnicity (n)			
White/Caucasian, n (%)	14 (88%)	18 (72%)	
Black/African American, n (%)	1 (6%)	6 (28%)	
Hispanic, n (%)	1 (6%)	0 (0%)	
RHI y/n (%exposed)	7/9 (44%)	25/0 (100%)	
Exposure source, n (%)			
Football	6 (38%)	25 (100%)	
Combat veteran	0 (0%)	2 (8%)	
Rugby	2 (12%)	2 (8%)	
Soccer	0 (0%)	1 (4%)	
Wrestling	1 (6%)	0 (0%)	
Hockey	1 (6%)	3 (12%)	
Boxing	1 (6%)	2 (8%)	
Lacrosse	0 (0%)	1 (4%)	
RHI Exposure years (S.E.M.)	11(3)*	15(1)	P = .284
CTE Severity			
Low	0% (0)	48% (12)	
High	0% (0)	52% (13)	

Data are presented as mean (SEM) years for age at death and contact sports exposure and as # yes/# no (%) unless otherwise indicated

CTE: chronic traumatic encephalopathy; RHI: repetitive head impacts

^*^data was unavailable for one participant in this group

### Visualization of microvascular networks with tomato lectin^649^ fluorescence (Fig. 2)

**Fig. 2:**
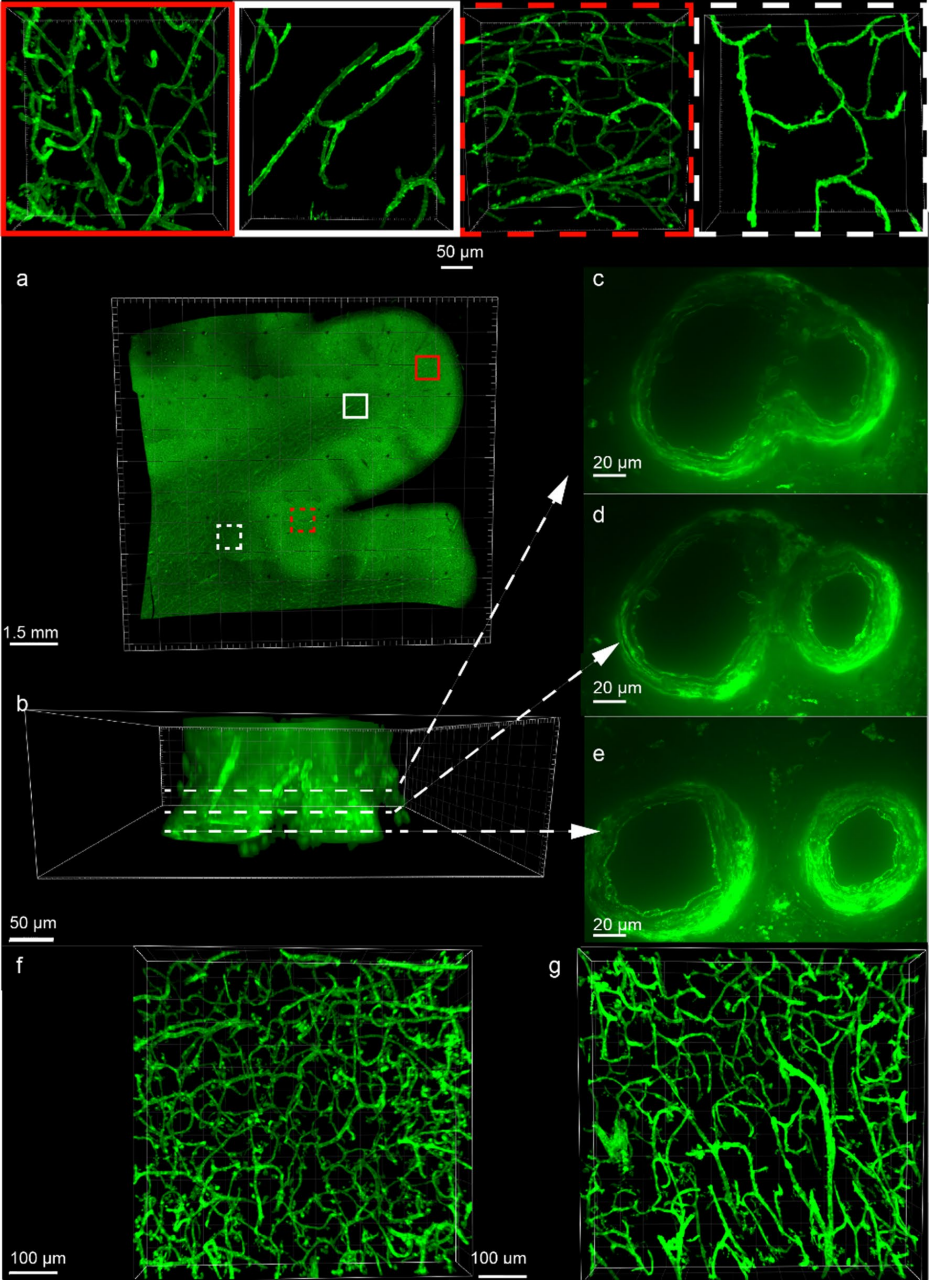
Images of blood vessels labeled with tomato lectin^649^ (green) in cleared and labeled PLP-fixed human DLF tissue. **a** 200 µm thick DLF tissue section after clearing and staining with tomato lectin^649^, imaged at 4x. Overlaid squares represent the areas sampled per case: gray matter crest (red), white matter crest (white), gray matter sulcus (red dashed), and white matter sulcus (white dashed). 20 × images of each region are shown at top, dimension of each 347 × 336 × 91 µm. **b** 20 × image of a branching blood vessel and 60 × optical slices taken at 9 µm (**c**), 27.6 µm (**d**), and 35.4 µm into the tissue slice (**e**).** f** and** g** both show 20 × images of passively cleared and tomato lectin^649^-stained DLF tissue from different cases in the gray matter sulcus. A CTE case with high blood vessel fraction volume and branch density is shown in (**f**), with fraction volumes of 0.10 and and branch densitiy of 1500 branches/mm^3^.** g** shows an image stack from a non-CTEcontrol with lower vessel fraction volume (of 0.069) and branch density (980 branches/mm^3^). Each image stack is 81 µm thick

SHIELD tissue treatment, passive delipidation, and staining was successful for visualizing blood vessels in three dimensions. The patterns of tomato lectin^649^ fluorescence were distinctive in the gray and white matter, with many branching curving vessels in the gray matter and more linear vessels in the white matter with fewer branches (Fig. 2a, top). As seen in Fig. 2b–e, a branching blood vessel visualized in a single plane can appear as either one (Fig. 2c), an individual vessel with multiple lumina (Fig. 2d), or as two vessels (Fig. 2e) depending on the focal plane. Imaging a 200 µm tissue section allows visualization of the vessel in 3-D and captures vascular branching points and vascular network structure.

### CTE is associated with increased vascular branching in the gray matter sulcus

Vascular density and blood vessel branching could be qualitatively observed in image stacks of at least 100 µm thickness. In general, blood vessel density was greater, and there were more branching blood vessels in CTE compared to control cases (Fig. 2f–g). Blood vessel branch density was increased in the DLF gray matter sulcus of the CTE group compared to the control group. Means of blood vessel branch densities were compared using an ANCOVA with the age of death as a covariate. The CTE group had a 24% higher branch density than the controls. Gray matter sulcus branch density of the CTE group (1180 ± 40 branches/mm^3^) was significantly higher than controls (950 ± 50 branches/mm^3^), *p* = 0.0011. The statistical power for this difference was 0.9963. There were no significant differences in blood vessel branch density among groups in the white matter. The significance between groups was unaffected whether or not CAA, arteriolosclerosis, and atherosclerosis were included as covariates.

### Vascular branching and density are greater in the sulcus than the crest in CTE but not controls

**Fig. 3:**
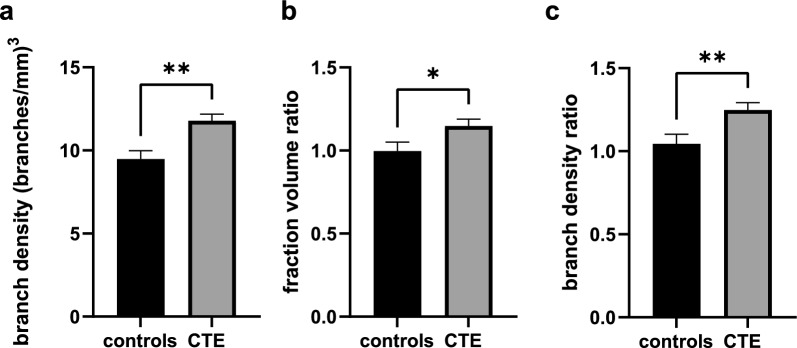
Quantification of DLF vascular branching and coverage. Differences between groups were established by ANCOVA with means adjusted for evaluation at mean age of death = 64.2 years and CAA = 0.3659. The gray matter branch density and fraction volume ratios were calculated by dividing the branch density or fraction volume of the sulcus by the branch density or fraction volume of the crest. **a** Gray matter sulcus branch density, **b** Gray matter branch density ratio **c** Gray matter fraction volume ratio. *p < 0.05, **p < 0.01, ***p < 0.001. CTE *n* = 25, controls *n* = 15

We compared vascular density and branching in the sulcal depth to that in the gyral crest by dividing the mean branch density and fraction volume in the sulcus by the measurements in the crest for each case. The group means of the calculated ratio were compared between groups with ANCOVA using the age of death as a covariate. The ratio of gray matter blood vessel branch density in the sulcus compared to the crest was 1.25 ± 0.04 for the CTE group and 1.04 ± 0.06 for the control group, a significant difference (*p* = 0.0076) with a power of 0.9613, (Fig. 3b).

The CTE group also had a higher ratio of sulcus:crest fraction volume than controls. Controls showed a fraction volume of 1.00 ± 0.05. The CTE group had a significantly greater fraction volume ratio, 1.15 ± 0.04, *p* = 0.0336, (Fig. 3c). The statistical power of this difference was 0.8445. No significant differences between CTE and controls were seen with the blood vessel branch density ratios or fraction volume in the white matter. Arteriolosclerosis and atherosclerosis were not significant predictors of blood vessel fraction volume ratio or blood vessel branch density ratio.

### CTE is associated with greater vascular coverage in the absence of CAA

**Fig. 4:**
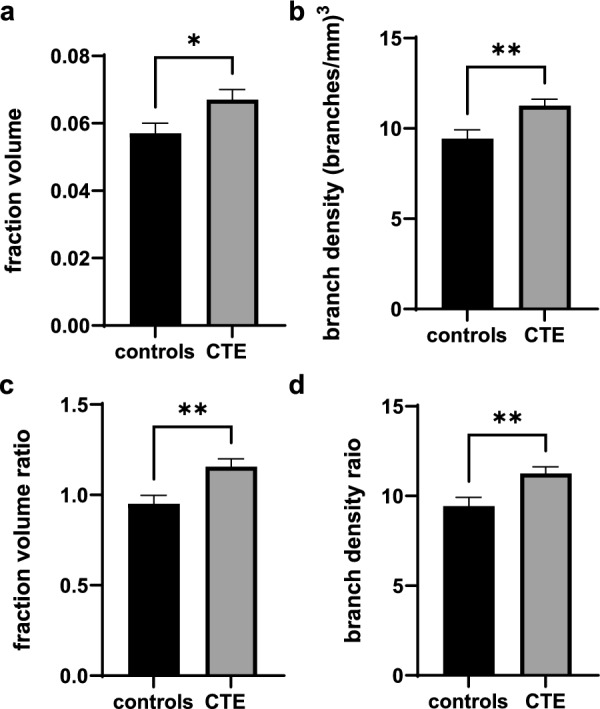
Quantification of DLF vascular branching and coverage in cases without CAA. Differences between groups were established by ANCOVA with means adjusted for evaluation at mean age of death = 61.4 years. The gray matter branch density and fraction volume ratios were calculated by dividing the branch density or fraction volume of the sulcus by the branch density or fraction volume of the crest. **a** vessel fraction volume in the gray matter sulcus, **b** vessel branch density in the gray matter sulcus, **c** gray matter fraction volume ratio, and **d** gray matter branch density ratio. *p < 0.05, **p < 0.01, ***p < 0.001. CTE *n* = 17, controls *n* = 13

When cases with CAA were removed from the analysis, CTE cases had a higher gray matter sulcus fraction volume than control cases. With our full dataset, changes in fraction volume were driven primarily by CAA (*p* = 0.007 for gray matter sulcus fraction volume), as calculated via ANCOVA with the age of death as a covariate comparing groups with and without CAA. As CAA is characterized by amyloid-beta deposits in the vessel walls, it is likely that there is a greater fraction volume detected in CAA cases because of the thickened vessel walls. To eliminate the effect of CAA on comparisons between CTE and controls, cases with CAA were eliminated from the analysis such that *n* = 13 control cases without CAA and *n* = 17 CTE cases without CAA. With this subset of data, there was a 17% increase (*p* = 0.0282) in gray matter sulcus vessel fraction volume of CTE cases compared to controls (Fig. 4a). Removing cases with CAA did not take away statistical significance from any of the other comparisons of measured vascular variables (Fig. 4b–d).

### P-tau pathology correlates with increased DLF gray matter vascular density

We compared p-tau pathology with blood vessel branch density and fraction volume in the gray matter sulcus. We pooled the control and CTE groups and conducted multiple linear regression with the age of death and CTE status as a covariate, p-tau staining density as the independent variable, and branch density or fraction volume as the dependent variable. P-tau staining density in the sulcus correlated positively with sulcal gray matter fraction volume (Table 3). The correlation coefficient between the two variables was 0.472 with a corresponding *p*-value of 0.013 (indicated in bold). Age at death did not significantly predict gray matter fraction volume or branch density. The correlation between AT8 staining density and gray matter sulcus fraction volume was not driven by CTE status.

**Table 3:** Multiple linear regression model demonstrating that p-tau pathology (AT8 staining density) in the DLF sulcus correlates with vascular branch density and fraction volume. β = standardized Beta, SE = standard error

	GM sulcus fraction volume			GM sulcus branch density (branches/mm^3^)		
	β	SE	*p*-value	β	SE	*p*-value
AT8 density	0.472	6 × 10^–8^	**0**.**013**	0.203	9 × 10^–12^	0.284
age at death	0.232	2 × 10^–4^	0.189	0.072	2 × 10^–8^	.695
CTE	0.139	6 × 10^–3^	0.445	0.384	8 × 10^–7^	0.053

## Discussion

**Fig. 5:**
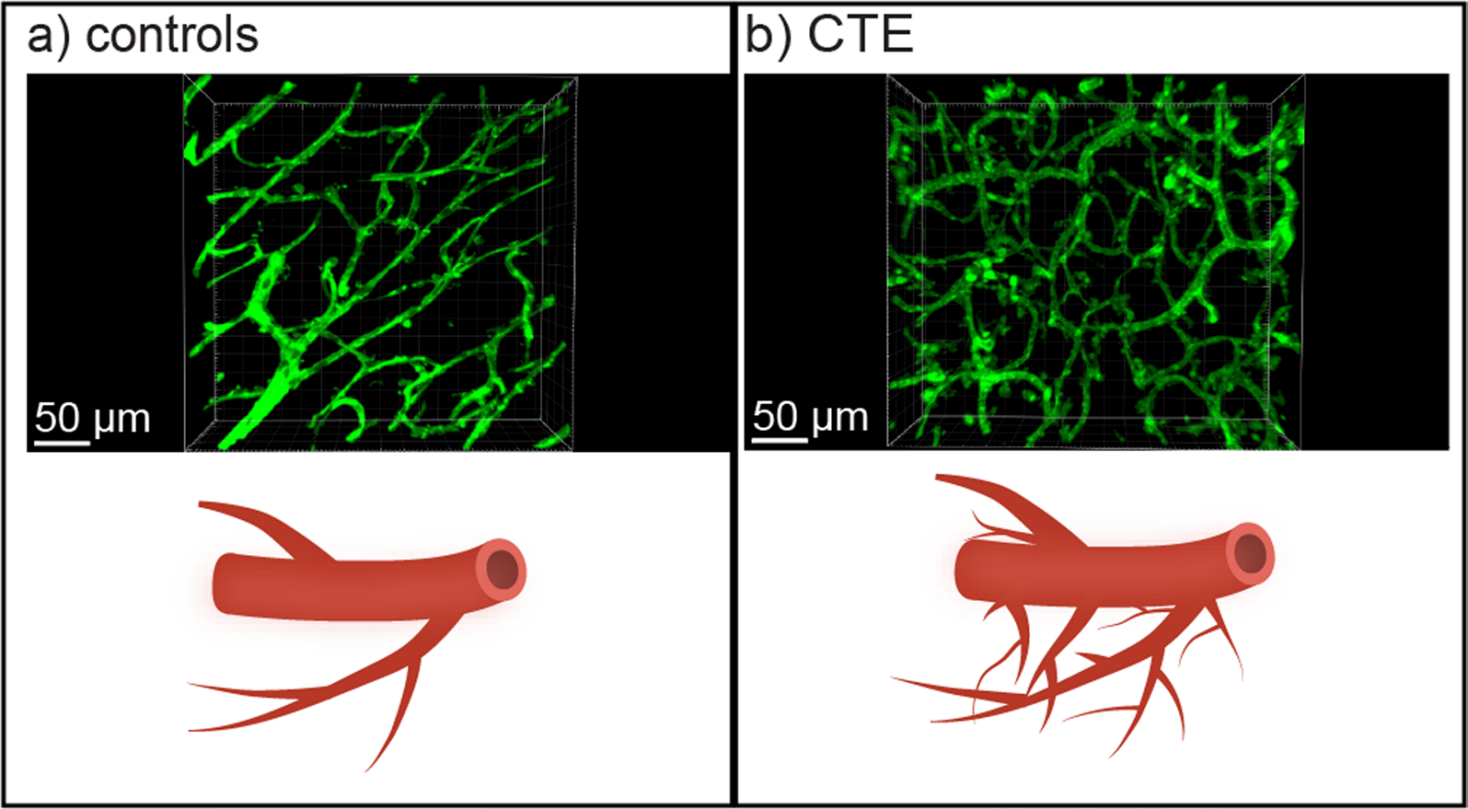
Vascular branching and coverage in the setting of repetitive head impacts and CTE. Scheme for the association between vascular branching, RHI, and CTE in the DLF gray matter sulcus. Illustrations representing **a** controls and **b** CTE cases. Each illustration is paired with a 347 µm × 335 µm × 81 µm fluorescent tomato lectin^649^ (green) image stack of DLF gray matter sulcus tissue from a representative case. As reflected in our data, **b** shows a significantly higher branch density in the sulcus than **a**

Using SHIELD postfixation and passive delipidation, we found increased vascular branching and coverage in the gray matter sulcus in CTE and increased vascular branching and coverage in the sulcus compared to the crest in CTE. We also found that vascular coverage in the sulcus correlated with p-tau pathology. These findings suggest that there is increased vascular branching and coverage in regions with p-tau pathology in CTE. Figure 5 outlines vascular branching in the DLF gray matter sulcus as seen in our measurements.

The increased vessel branching and coverage found in DLF gray matter sulcus in CTE may be explained by prior angiogenesis. Cortical neovascularization may be a chronically active process that occurs in parallel with CTE p-tau pathology development and progression. The combination of direct injury to vascular endothelial cells, inflammation, and secondary tissue hypoxia creates a proangiogenic environment. Chronic neuroinflammation and tissue hypoxia due to compromised vasculature results in the release of proangiogenic proteins [3, 9, 25, 59, 66, 70]. Neoangiogenic processes are activated by inadequate tissue oxygenation and hypoxia to increase vascular coverage [65]. RHI itself may also induce angiogenesis mechanically [29, 35]. Military veterans that have mTBI from blast exposure have increased serum concentrations of vascular endothelia growth factor (VEGF-A), indicating a correlation between mTBI and pro-angiogenic signaling [55]. Molecular markers of vascular injury have also been observed in tissue from the biorepositories used in this study [38]. The observed increased vessel branch density in CTE cases compared to controls supports prior angiogenesis due to the branching progression of new vessel formation [15, 23, 65]. If the increased branching observed in CTE cases is due to angiogenesis, it is likely to have occurred in the distant past. None of our cases showed positive staining for Ki67, a marker of cellular proliferation (Additional file 3: Supplementary Fig. S1). Another putative line of evidence for prior angiogenesis is increased vascular coverage, measured as increased vessel fraction volume. The ratios of gray matter branch density and fraction volume were greater than 1 in CTE but below 1 in controls, suggesting that sulcus-specific increased vascular branching and coverage are associated with CTE. Previous antemortem MRI work has highlighted the susceptibility of blood vessels in the gray matter sulci to damage as a result of impact forces [39]. In CTE, gray matter sulci have been exposed to repetitive forces and secondary hypoxia from vessel damage that creates a proangiogenic environment. Cortical neovascularization may be a chronically active process that occurs in parallel with CTE p-tau pathology development and progression.

The observed increase in vessel branching and fraction volume in the sulcus compared to the crest may also be due to atrophy of surrounding brain tissue. Lower volumes of gray matter and white matter in the cortical sulci have been observed in individuals exposed to TBI compared to unexposed controls [22]. Individuals with postmortem neuropathologically diagnosed CTE also had more atrophy in the frontal cortex, visualized with antemortem MRI, compared to controls [6]. It is possible that there are more vessel branches per unit volume in CTE cases compared to controls, because there is less surrounding tissue to contribute to the total unit volume.

There was a correlation between p-tau density with increased vascular fraction volume in the gray matter sulci of individuals with exposure to RHI. This correlation suggests potential prior vascular remodeling and/or tissue atrophy of high p-tau burden in CTE. Vascular remodeling and p-tau accumulation may be dependent or independent, as mechanical force alone is enough to cause tau mislocalization and expression of pro-angiogenic factors in vitro [14, 29].

Furthermore, this work demonstrates that microvascular networks in postmortem human brain tissue preserved for years in paraformaldehyde can be cleared, labeled, and confocally imaged in three dimensions. To the best of our knowledge, this is the first study using tissue clearing to probe human disease using banked, long-term fixed brain tissue with quantitative results.

## Limitations

There are several limitations to this work. Changes in vascular branching in the white matter were not detected by our techniques because the fraction volume and branch density of white matter vasculature was lower and dominated by a smaller number of larger vessels. Imaging greater volumes of tissue would be needed to robustly detect white matter changes. While the technique used here is well suited to the microvasculature, lower magnification optical coherence tomography is better for visualization of larger white matter vessels [72, 77]. Moreover, there are unavoidable drawbacks to using postmortem human tissue such as varying tissue extraction and fixation conditions, small sample size, comorbidities, clinical data collection, and convenience sampling. Although we adjusted for age, confounding by age may exist because the age ranges do not entirely overlap. We addressed comorbidities by statistically determining which comorbidities affected our data and included them in the models as covariates. The diversity of the study population was constrained by the tissue available in the UNITE and PTSD brain banks. Future work should include participants of all genders and races as more tissue is available.

## Future directions

The results of this research suggest several avenues for future inquiry. The sulcus-to-crest ratios of vascular branching and fraction volume are worth further exploration for possible use in clinical diagnosis. In addition, since small vascular disease or arteriolosclerosis is associated with dementia in CTE [7, 26], future work will help determine whether the vascular changes found in this work are also associated with cognitive impairment. The SHIELD tissue post-fixing and clearing techniques used here could also be combined with immunofluorescence to evaluate multiple markers in future studies.

## Conclusions

We used tissue clearing to measure and compare vascular coverage and branching in postmortem brain tissue from individuals with CTE and controls. Our work suggests an association between vascular branching and density and CTE. Sulcus-specific vascular coverage increases also correlated positively with p-tau pathology providing further evidence to suggest that microvascular abnormalities and p-tau pathology might be mechanistically linked in CTE.

## Supplementary Information

**Additional file 1.** Causes of death and comorbidities of brain donors.

**Additional file 2.** Full data set of clinical, neuropathological, and measured vascular data.

**Additional file 3.** Examples of negative Ki67 immunohistochemical staining. Images Ki67 staining and DAPI on cases with a) No CTE or RHI exposure, b) No CTE with RHI exposure, c) Low CTE, and d) High CTE.

## Data Availability

The raw images analyzed in the current study are available from the corresponding author upon reasonable request. The dataset quantified from the images in the current study is available online (Additional file 2).
