# Supplementary material for: Three dimensional evaluation of cerebrovascular density and branching in chronic traumatic encephalopathy
Source: Acta Neuropathol Commun. 2023 Jul 25;11:123. doi: 10.1186/s40478-023-01612-y (PMC10369801; doi:10.1186/s40478-023-01612-y)
Supplement: Supplementary file 1 — Additional file 1. Causes of death and comorbidities of brain donors. [file 40478_2023_1612_MOESM1_ESM.docx]

**Table S1**. Causes of death and comorbidities of brain donors.

|  | **controls** | **CTE** |
| --- | --- | --- |
| **cause of death n, (%)** |  |  |
| cardiovascular | **8 (50%)** | **7 (28%)** |
| neurodegenerative | **1 (6%)** | **8 (32%)** |
| accidental overdose | **1 (6%)** | **2 (8%)** |
| suicide | **3 (19%)** | **2 (8%)** |
| motor vehicle accident | **0 (0%)** | **0 (0%)** |
| pneumonia | **1 (6%)** | **0 (0%)** |
| liver disease | **1 (6%)** | **1 (4%)** |
| injury | **0 (0%)** | **1 (4%)** |
| cancer | **0 (0%)** | **1 (4%)** |
| other | **1 (6%, mucus plug)** | **3 (12%)** |
| **vascular comorbidities n, (%)** |  |  |
| atherosclerosis | **3 (19%)** | **4 (16%)** |
| arteriolosclerosis | **12 (75%)** | **15 (60%)** |
| cerebral amyloid angiopathy | **3 (19%)** | **8 (32%)** |
| **neurodegenerative comorbidities (n)** |  |  |
| TDP-43 | **1 (6%)** | **10 (40%)** |
| dementia | **2 (12%)** | **8 (32%)** |
